# Amitriptyline Dependence and Its Associations: A Case Report and Literature Review

**DOI:** 10.1155/2021/6647952

**Published:** 2021-01-29

**Authors:** T. Umaharan, S. Sivayokan, S. Sivansuthan

**Affiliations:** ^1^Senior Registrar in Psychiatry Teaching Hospital, Jaffna, Sri Lanka; ^2^Consultant Psychiatrist, Teaching Hospital, Jaffna, Sri Lanka; ^3^Consultant in Internal Medicine Teaching Hospital, Jaffna, Sri Lanka

## Abstract

Amitriptyline, the second antidepressant invented next to imipramine, is indicated in many psychiatric conditions as well as for some organic disorders. The drug acts by increasing the availability of monoamines in the central nervous system postsynaptic clefts. Amitriptyline has long been suspected for abusive potential based on a few case reports, and the reports add evidence in favor of the hypothesis. This case report brings such material to the arena of evidence and discusses the probable mechanisms by which patients turn to abusing and be addicted to the drug. The article also argues matters associated with drug dispensing that might raise the risk of misuse of the drug, especially in countries where strict legislation for accessibility of prescribed drugs is not in practice.

## 1. Introduction

Amitriptyline is a tricyclic antidepressant and is used to treat depression, anxiety disorders, posttraumatic stress disorder (PTSD), insomnia, somatoform disorders, premenstrual dysphoric disorder, nocturnal enuresis, migraine, and neuropathic pain [1–3]. It blocks the reuptake of norepinephrine and serotonin and thus increases the availability of these neurotransmitters in the central nervous system (CNS) [1]. The maximum dose per day of amitriptyline is 300 mg, and this is given in divided doses to prevent/minimize adverse effects [2, 3]. The drug has antagonistic action at histaminic-1, alpha-1-adrenergic, and muscarinic cholinergic receptors. Those antiadrenergic and antimuscarinic properties bring about the troublesome adverse effects (hypotension, tachycardia, blurring of vision, urinary retention, constipation, dry mouth, sexual dysfunction) of the drug [1–3]. Amitriptyline also blocks voltage sensitive sodium channels in the heart and brain. In overdose, this action leads to arrhythmia, seizures, coma, and death [1].

The side effects, toxicity, and lethality in overdose, the risk of switch to manic state in predisposed individuals and the invention of selective serotonin reuptake inhibitors (SSRIs), another class of antidepressants with a better safety profile, made amitriptyline a less preferable medication [2]. However, the drug still has its role as a second line treatment for depression and nonpsychiatric conditions [1–3]. Amitriptyline also lowers the seizure threshold, and as such, in higher doses or intoxication, amitriptyline causes seizures [1, 4, 5]. Though it is generally considered that the drug does not possess abusive or addictive properties, there are a few case reports which suggest amitriptyline may exert such effects in susceptible individuals, i.e., patients with a history of another psychoactive substance abuse [6–10].

## 2. Case History

A 39-year-old married father of two videographer by profession, and who getting treatment for type II diabetes mellitus (DM), was admitted to a divisional hospital (Chankanai, Jaffna) in northern Sri Lanka, as he was found unconscious and suffering from seizure attacks. The patient was then transferred to Teaching Hospital Jaffna, as his seizure attacks could not be controlled with the treatment given at the divisional hospital. The patient was immediately admitted to the intensive care unit (ICU), a diagnosis of status epilepticus was made, and he was anesthetized in view of controlling his seizures. Within the next seven hours, the patient had 12 episodes of seizures. His electroencephalogram (EEG) and noncontrast computerized tomography (CT) scan of the brain, cerebrospinal fluid, liver enzymes, serum electrolytes, random blood sugar, and other routine biochemicals were well within normal limits. His electrocardiogram (ECG) showed sinus tachycardia. Opinion from a neurology team was sought, and it was favored against the diagnosis of epilepsy. The patient's wife gave a history that further enlightened the clinical picture.

Accordingly, the patient was regularly using alcohol for many years. One day, he consulted a surgeon in the private sector as he developed a single episode of haematemesis. The surgeon pointed out the reason behind the problem and advised him to go for total abstinence from alcohol. The surgeon also prescribed a low dose of alprazolam (0.5 mg nocte) to support his sleep without alcohol. In about a year's time, the patient was again presented to the surgeon by his family with a different complaint—“uncontrolled use of alprazolam”. At that point, the surgeon referred the patient to a psychiatrist. The psychiatrist advised the patient to tail off alprazolam and started treating him with 25 mg of amitriptyline, to be taken at night, in view of supporting his sleep problems. In the subsequent visit, the patient was complaining of poor sleep, and the dose of amitriptyline was gradually increased to 75 mg nocte. The patient felt better with that dose and stopped visiting the psychiatrist.

Since then, the patient started a kind of self-medication. He gradually increased the dose of amitriptyline to 250-300 mg at night as he needed more tablets to have good quality sleep and used 100–250 mg in daytimes to prevent dysphoria and restlessness, which were experienced by him in the absence of amitriptyline. He could not control taking amitriptyline in excess doses despite its regular side effects, which include constipation. In addition, from time to time, he engaged in binge ingestion of amitriptyline when he wanted to experience a “high” in his mood. In such circumstances, the number of tablets even reached to 25-30 per occasion (625–750 mg). These binges were associated with one or two seizure attacks, for which he or his family did not seek medical attention. For the past one year, the patient developed a progressive decline in his functionality, and he became dependent on amitriptyline. On the day of admission, the patient ingested about 30 tablets around 3 pm. Soon, the patient developed loss of consciousness and seizures.

After two days, anesthesia was withdrawn, and the patient was observed for the possibility of reappearance of seizure attacks for the next few hours. No more seizures were observed; however, he was continuously found to be confused. The patient was then transferred to an inpatient medical facility. In the medical ward, the patient soon became agitated, overtalkative, euphoric, disinhibited, and slept poorly. The diagnosis of delirious mania or hyperactive delirium as a result of the late effect of amitriptyline intoxication was made, and then he was started on effective doses of haloperidol and quetiapine. His symptoms responded well to haloperidol 6 mg tds and quetiapine 100 mg nocte. After recovery, the patient was assessed for the possibility of underlying depression, anxiety states, suicidal behavior, and any other psychiatric morbidities, and all were negative. However, he fulfilled the dependence criteria for amitriptyline. As per request, the family brought the drug pack for inspection, and it was found to contain about 500 tablets of 25 mg strength amitriptyline.

He was discharged from the hospital after two weeks on quetiapine 200 mg nocte, enrolled in an abstinence programme for amitriptyline dependence, taught sleep hygiene measures and interpersonal social rhythm therapy with the aim of assisting his routine sleep and other day-to-day activities, supported to reengage in his profession and to practice regular physical exercise, and was placed on assertive follow up care to monitor for and to prevent relapse.

## 3. Discussion

Patients who are treated with amitriptyline for long-term may show discontinuation syndrome upon withdrawal of the drug. This commonly manifests as flu-like symptoms (chills, myalgia, headache, nausea, excessive sweating), insomnia, excessive dreaming, and occasionally, movement disorders, mania, and cardiac arrhythmia [2].However, this patient's symptoms did not fit into this cluster of symptoms. Therefore, the possibility of discontinuation syndrome is least likely.

The diagnosis of amitriptyline dependence was made as the patient showed dependence features to the drug (tolerance, withdrawal symptoms, craving, continuation, and negligence of duty), and they were present for a period of more than one year [11].Features of dependence are broadly categorized into two classes: psychological and physiological [2] .Though the patient displayed both these types of symptoms, the psychological symptoms outweighed the physiological ones.

This case shines light on the long-lasting question of scientists whether amitriptyline holds abusive and dependency potential. Amitriptyline's abusive and addictive properties may result from its euphoric and sedative effects, similar to alcohol [1], and its (psycho) stimulant effect, as evident from this patient's urge to have “highs” that was “satisfied” with binging (overdose) of the drug [10]. Richelson [12] argues that anticholinergic and antihistaminic effects of tertiary tricyclics may underlie their abusive liability. Therefore, it could be argued that the antihistaminic and anticholinergic properties of amitriptyline act synergistically, resulting in its abusive tendency. However, there are case studies which divulge that the antihistaminic and anticholinergic properties of drugs may result in stimulant, euphoric, and/or psychedelic effects that might lead users to abuse these drugs [13–17]. These articles suggest that the stimulant and euphoric properties of amitriptyline may underlie the probable mechanism by which the drug causes addiction and dependence.

The other important concern the case highlights is that individuals with substance use disorders might carry a higher risk for amitriptyline dependence. Psychiatrists and other physicians who use amitriptyline in their practice should be prudent and keep a watchful eye on this long-term risk of the drug. In addition, this case discloses a deficient area of drug prescription and free accessibility of medications which is highly prevalent in Sri Lanka and may be in other countries as well, where no strict legislations govern the purchase of prescribed medications. The patient purchased the medication with an old prescription which was written about one and a half years ago by his psychiatrist. The case supports the argument that there should be a strict protocol to be adhered in accessing prescribed medications.

## 4. Conclusions

1. Amitriptyline shows the potential to cause dependence syndrome in vulnerable individuals
2. A clear protocol should be implemented when dispensing medications from pharmacy to patients
